# Salvianolic Acid A Protects the Kidney against Oxidative Stress by Activating the Akt/GSK-3*β*/Nrf2 Signaling Pathway and Inhibiting the NF-*κ*B Signaling Pathway in 5/6 Nephrectomized Rats

**DOI:** 10.1155/2019/2853534

**Published:** 2019-03-18

**Authors:** Hong-feng Zhang, Jia-hong Wang, Yan-li Wang, Cheng Gao, Yan-ting Gu, Jian Huang, Jin-hui Wang, Zhou Zhang

**Affiliations:** ^1^Department of Physiology, School of Life Science and Biopharmaceutics, Shenyang Pharmaceutical University, Shenyang 110016, China; ^2^Department of Medicinal Chemistry and Natural Medicine Chemistry, State-Province Key Laboratories of Biomedicine-Pharmaceutics of China, Harbin Medical University, Harbin 150081, China; ^3^Shenzhen Honghui Biopharmaceutical Co., Ltd. Shenzhen 518000, China

## Abstract

Salvianolic acid A (SAA) is a bioactive polyphenol extracted from *Salviae miltiorrhizae* Bunge, which possesses a variety of pharmacological activities. In our previous study, we have demonstrated that SAA effectively attenuates kidney injury and inflammation in an established animal model of 5/6 nephrectomized (5/6Nx) rats. However, there has been limited research regarding the antioxidative effects of SAA on chronic kidney disease (CKD). Here, we examined the antioxidative effects and underlying mechanisms of SAA in 5/6Nx rats. The rats were injected with SAA (2.5, 5, and 10 mg·kg^−1^·d^−1^, ip) for 28 days. Biochemical, flow cytometry, and Western blot analyses showed that SAA significantly increased the activities of total superoxide dismutase (T-SOD), glutathione peroxidase (GPx), and catalase (CAT) and lowered the levels of malondialdehyde (MDA), reactive oxygen species (ROS), and NADPH oxidase 4 (NOX-4) in a dose-dependent manner in 5/6Nx rats and in H_2_O_2_-induced HK-2 cells *in vitro*. Moreover, SAA enhanced the activation of the protein kinase B/glycogen synthase kinase-3*β*/nuclear factor-erythroid-2-related factor 2 (Akt/GSK-3*β*/Nrf2) signaling pathway in a dose-dependent manner and subsequently increased the expression of heme oxygenase-1 (HO-1) in the kidney of 5/6Nx rats, which were consistent with those obtained in H_2_O_2_-induced HK-2 cells *in vitro* shown by Western blot analysis. Furthermore, SAA significantly increased the expression of intranuclear Nrf2 and HO-1 proteins compared to HK-2 cells stimulated by LPS on the one hand, which can be enhanced by QNZ to some extent; on the other hand, SAA significantly lowered the expression of p-NF-*κ*B p65 and ICAM-1 proteins compared to HK-2 cells stimulated by H_2_O_2_, which can be abrogated by ML385 to some extent. In conclusion, our results demonstrated that SAA effectively protects the kidney against oxidative stress in 5/6Nx rats. One of the pivotal mechanisms for the protective effects of SAA on kidney injury was mainly related with its antioxidative roles by activating the Akt/GSK-3*β*/Nrf2 signaling pathway and inhibiting the NF-*κ*B signaling pathway.

## 1. Introduction

Chronic kidney disease (CKD) has high morbidity and mortality and has become a serious public health concern worldwide [1–3]. Prevalence is estimated to be 8–16% worldwide [2]. According to a systematic analysis for the 2015 Global Burden of Disease Study, the mortality of CKD increased by about 30% and attributable mortality and disability-adjusted life-years also significantly increased between 2005 and 2015, which was due to low glomerular filtration rates [4]. Two important features of CKD are oxidative stress and inflammation, which are inseparably linked and play key roles in driving the development and progression of CKD and other complications [5]. CKD patients usually have multiple cardiovascular risk factors including hypertension, diabetes mellitus, and dyslipidemia associated with oxidative stress, which can induce inflammatory processes and accelerate the progression of CKD [6]. Thus, inhibiting oxidative stress may delay the progression of CKD. 5/6 nephrectomized (5/6Nx) rats were used to model chronic kidney disease and have frequently been used to study the progression of CKD [7–9]. During CKD, reactive oxygen species (ROS) were elevated in the proximal tubule and promoted lipid peroxidation and the progression of CKD [10]. HK-2 cells stimulated *in vitro* with H_2_O_2_ were used to identify antioxidative effects in the kidney [11–13].

Oxidative stress is a condition, in which the balance between the oxidative and antioxidative systems is destroyed and excessive ROS are produced. Researches have shown that oxidative stress, increase of nicotinamide adenine dinucleotide phosphate (NADPH) oxidase, and decrease of antioxidant enzymes are involved in the pathogenesis of CKD [14–18].

The nuclear factor-erythroid-2-related factor 2 (Nrf2) is a transcription factor that can regulate genes encoding antioxidant and cytoprotective proteins, such as catalase (CAT), superoxide dismutase (SOD), heme oxygenase-1 (HO-1), and glutathione peroxidase (GPx) [5, 19, 20]. In basal conditions, Nrf2 is kept as an inactive complex and bound to Kelch-like ECH-associated protein 1 (Keap1) in the cytoplasm, which can facilitate the ubiquitination of Nrf2. Once Nrf2 is activated, it translocates into the nucleus and induces the transcription of its downstream genes when exposed to oxidative stress [5, 12]. Previous studies have suggested that the activity of Nrf2 and the expression of its downstream proteins were markedly reduced in the kidney in CKD animals [5, 9, 15, 21, 22]. Furthermore, evidence recently showed that NF-*κ*B directly represses Nrf2 signaling at the transcription level by depriving CREB-binding protein (CBP) from Nrf2 or facilitating recruitment of histone deacetylase 3 (HDAC3) to MafK (one of the small Maf proteins) [23, 24]. However, the latent crosstalk between these two signaling pathways needs to be elucidated in more detail during CKD. Nrf2 can be activated via phosphorylation of its threonine or serine residues by upstream kinases, such as mitogen-activated protein kinases (MAPK), protein kinase C (PKC), casein kinase-2, phosphatidylinositol-3-kinase/protein kinase B (PI3K/Akt), and the endoplasmic reticulum enzyme PERK [5, 25, 26]. It has been reported that Puerarin induces the nuclear translocation of Nrf2 for protection of cells against oxidative stress through activating the PI3K/Akt/GSK-3*β* signaling in PC12 cells exposed to lead [27]. Furthermore, another study suggested that fenofibrate prevents the progress of diabetic nephropathy via upregulating FGF21 and activating the PI3K/Akt/GSK-3*β*/Fyn/Nrf2 pathway [28]. However, the exact mechanism of the Akt/GSK3*β*/Nrf2 signaling pathway during CKD remains not well understood.

Salvianolic acid A (SAA) is a bioactive polyphenol extracted from the root of *Salviae Miltiorrhizae* (Danshen), a versatile traditional Chinese herbal medicine. Studies have shown that SAA is a multitarget agent possessing a variety of pharmacological activities, such as antioxidant [29], anti-inflammatory [30], antifibrotic [31], antiplatelet, and antithrombotic properties [32]. In our previous study, we have demonstrated that SAA attenuates kidney injury and inflammation in 5/6Nx rats [33]. Additionally, another study reported that SAA exhibits renoprotection in doxorubicin-induced nephropathy through its roles in antioxidation, anti-inflammation, and amelioration of podocyte injury [34]. Furthermore, SAA has been reported to protect against oxidative stress via activating the Nrf2/HO-1 signaling in retinal pigment epithelial (RPE) cells [20].

However, up to now, the antioxidative effects of SAA in CKD remain poorly understood. In our study, we evaluated the antioxidative effects and molecular mechanisms of SAA in an established model of 5/6Nx rats and further confirmed the results in H_2_O_2_-induced HK-2 cells *in vitro*.

## 2. Materials and Methods

### 2.1. Materials

SAA (purity≧98% by HPLC) was provided by the laboratory of Dr. Jin-hui Wang (Shenyang Pharmaceutical University). The antibodies used in this study were as follows: anti-p-Nrf2 (abs137005a, Absin), anti-Nrf2 (16396-1-AP, Proteintech), anti-HO-1 (10701-1-AP, Proteintech), anti-NADPH oxidase 4 (NOX-4) (14347-1-AP, Proteintech), anti-ICAM-1 (#60299-1-lg, Proteintech), anti-*β*-actin (66009-1-lg, Proteintech), anti-p-Akt (#4060, CST), anti-Akt (#4691, CST), anti-p-GSK-3*β* (#5558, CST), anti-GSK-3*β* (#12456, CST), anti-p-NF-*κ*B p65 (#3033, CST), anti-NF-*κ*B p65 (#8242, CST), and anti-histone H3 (#4499, CST). The anti-rabbit and anti-mouse secondary antibodies were also obtained from CST.

### 2.2. *In Vivo* Experiments

#### 2.2.1. Animals and Experimental Design

Adult male Sprague-Dawley (SD) rats weighing 200–250 g were purchased from the Center for Experimental Animals of Shenyang Pharmaceutical University. Rats were maintained under a 12 h light-dark cycle and fed standard rodent chow and tap water ad libitum in an environmentally controlled room. Rats were acclimated for 1 week and then randomly divided into five groups as follows: group 1: the 5/6 nephrectomized group (5/6Nx; *n* = 10), in which the upper and lower poles of the left kidney were ablated and subsequently, the right unilateral nephrectomy was performed 1 week later; groups 2, 3, and 4: SAA-treated 5/6Nx rats were injected with 2.5 mg/kg, 5 mg/kg, and 10 mg/kg SAA (5/6Nx+SAA (2.5, 5, and 10 mg/kg), respectively, ip; *n* = 10); group 5: the control group (*n* = 10) was subjected to sham operation. Surgery was performed under anesthesia with administration of chloral hydrate solution (350 mg/kg, ip). All animal experiments were carried out as per the National Institutes of Health Guide for the Care and Use of Laboratory Animals, as well as according to the guidelines of animal handling of our university authority.

#### 2.2.2. Activity of Antioxidant Enzymes and Lipid Peroxidation Analysis

After 28 days of administration, all rats were sacrificed with intraperitoneal injection of chloral hydrate (350 mg/kg) and the kidney tissues were rapidly removed (isolated kidney tissue from adipose tissue). Kidney tissues were homogenized in PBS on ice followed by centrifugation at 12000 × *g* for 15 min at 4°C. The levels of total superoxide dismutase (T-SOD) activity, glutathione peroxidase (GPx) activity, catalase (CAT) activity, and malondialdehyde (MDA) in the supernatant were measured using commercial kits (Jiancheng Bioengineering Institute, Nanjing, China).

#### 2.2.3. ROS Assay

As a marker of oxidative stress, the level of ROS was determined by 2′,7′-dichlorofluorescein diacetate (DCFH-DA) (Jiancheng Bioengineering Institute, Nanjing, China) in renal homogenates according to the manufacturer's instructions. Kidney tissues were lysed in RIPA Lysis Buffer and homogenized on ice followed by centrifugation at 12000 × *g* for 15 min at 4°C. A volume of 50 *μ*L of the supernatant was placed in a 96-well plate and loaded with 10 *μ*L of DCFH-DA solution for 25 min at 37°C in the dark. The absorbance was measured using a microplate reader at 485/532 nm.

#### 2.2.4. Protein Extraction and Western Blot Analysis

Kidney tissues were homogenized in RIPA Lysis Buffer on ice, followed by centrifugation at 12000 × *g* for 15 min at 4°C. The BCA Protein Assay Kit (Beyotime Institute of Biotechnology, Shanghai, China) was used to measure protein concentration. Protein samples were separated using 10% SDS-PAGE gels and electrotransferred onto PVDF membranes and blocked with 5% skimmed milk in Tris-buffered saline for 2 h at room temperature. The membranes were incubated with primary antibodies (anti-p-Nrf2, anti-Nrf2, anti-HO-1, and anti-NOX-4 at 1 : 500 dilution; anti-p-Akt, anti-Akt, anti-p-GSK-3*β*, and anti-GSK-3*β* at 1 : 1000 dilution) overnight at 4°C and then incubated with horseradish peroxidase-conjugated anti-rabbit and anti-mouse secondary antibodies (1 : 5000 dilution). Proteins were visualized using ECL chemiluminescence agents (Wanlei Institute of Biotechnology, Shenyang, China) and developed by autoluminography.

### 2.3. *In Vitro* Experiments

#### 2.3.1. Cell Culture

Renal proximal tubule epithelial cells (HK-2 cells) were purchased from the American Type Culture Collection (ATCC) (Manassas, VA, USA). HK-2 cells were cultured in DMEM/F12 media containing 10% heat-inactivated FBS, 100 U/mL penicillin, and 100 *μ*g/mL streptomycin at 37°C in a 5% CO_2_-humidified incubator. The cells were treated with 300 *μ*mol/L H_2_O_2_ in the presence or absence of SAA at the indicated concentrations and incubation periods. SAA was dissolved in DMSO, and the final concentration of DMSO in media was under 0.1% in all groups.

#### 2.3.2. MTT Assay for Cell Viability

The 3-(4,5-dimethyl-2-thiazolyl)-2,5-diphenyl tetrazolium bromide (MTT) assay was used to detect cell viability. HK-2 cells were seeded in 96-well plates at 10^4^ cells/well. Following 24 h incubation, cells were pretreated with or without different concentrations of SAA (3, 10, and 30 *μ*mol/L) for 2 h and then coincubated with or without various concentrations of H_2_O_2_ for 24 h to assess the effects of SAA on cell viability in H_2_O_2_-induced HK-2 cells. For detecting the effects of ML385 or QNZ on cell viability, cells were treated with or without different concentrations of ML385 (an inhibitor of Nrf2, MedChemExpress) or QNZ (an inhibitor of NF-*κ*B, Selleck) for 24 h. Subsequently, the supernatant was removed and replaced with serum-free media and 20 *μ*L of MTT (5 mg/mL) and incubated for another 4 h. Next, the supernatant was removed and 100 *μ*L of DMSO was added to dissolve the formazan. The absorbance at *λ* = 490 nm was measured using a microplate reader.

#### 2.3.3. Activity of Antioxidant Enzymes and Lipid Peroxidation Analysis

HK-2 cells were seeded in 6-well plates at 2 × 10^5^ cells/well. After 24 h of incubation, cells were pretreated with or without different concentrations of SAA (3, 10, and 30 *μ*mol/L) for 2 h and then coincubated with or without H_2_O_2_ (300 *μ*mol/L) for 24 h. The cells were lysed in PBS by ultrasound on ice followed by centrifugation at 12000 × *g* for 15 min at 4°C. The levels of T-SOD, GPx, CAT activity, and MDA in the supernatants were measured using commercial kits (Jiancheng Bioengineering Institute, Nanjing, China).

#### 2.3.4. ROS Assay

Intracellular ROS was determined by 2′,7′-dichlorofluorescein diacetate (DCFH-DA) (Jiancheng Bioengineering Institute, Nanjing, China) according to the manufacturer's instructions. Briefly, HK-2 cells were seeded in 6-well plates at 2 × 10^5^ cells/well. After 24 h incubation, cells were pretreated with or without various concentrations of SAA (3, 10 and 30 *μ*mol/L) for 2 h and then coincubated with or without H_2_O_2_ (300 *μ*mol/L) for 12 h. Then HK-2 cells were loaded with 5 *μ*mol/L DCFH-DA for 30 min at 37°C in the dark and washed with PBS three times. The cells were sent for flow cytometry analysis (BD Biosciences) and measured using a 485/532 nm laser.

#### 2.3.5. Protein Extraction and Western Blot Analysis

For evaluating the effects of SAA on the Akt/GSK-3*β*/Nrf2 signaling pathway in H_2_O_2_-induced HK-2 cells, cells were pretreated with or without various concentrations of SAA (3, 10, and 30 *μ*mol/L) for 2 h followed by coincubation with or without H_2_O_2_ (300 *μ*mol/L) for 4 h to detect the expression of p-Akt, Akt, p-GSK-3*β*, GSK-3*β*, nuclear Nrf2, and cytosolic Nrf2 proteins or 24 h to detect the expression of HO-1 and NOX-4 before harvesting cells. To examine the relationship between the NF-*κ*B and Nrf2 signaling pathways, cells were pretreated with or without 10 *μ*mol/L ML385 or 0.5 *μ*mol/L QNZ for 10 h followed by coincubation with or without 30 *μ*mol/L SAA for 2 h and then stimulated with or without 300 *μ*mol/L H_2_O_2_ or 5 *μ*g/mL LPS until the cells were harvested. The nuclear and cytosolic proteins were prepared using a nuclear and cytoplasmic protein extraction kit (Beyotime Institute of Biotechnology, Shanghai, China). The cell total proteins were extracted in RIPA Lysis Buffer and homogenized on ice followed by centrifugation at 12000 × *g* for 15 min at 4°C. The BCA Protein Assay Kit (Beyotime Institute of Biotechnology, Shanghai, China) was used to measure protein concentrations. Protein samples were separated using 10% SDS-PAGE, and gels were electrotransferred onto PVDF membranes and blocked with 5% skimmed milk in Tris-buffered saline for 2 h at room temperature. The membranes were incubated with primary antibodies (anti-Nrf2, anti-HO-1, anti-NOX-4, and anti-ICAM-1 at 1 : 500 dilution; anti-p-Akt, anti-Akt, anti-p-GSK-3*β*, anti-GSK-3*β*, anti-p-NF-*κ*B p65, and anti-NF-*κ*B p65 at 1 : 1000 dilution; anti-histone H3 at 1 : 2000 dilution) overnight at 4°C and then incubated with horseradish peroxidase-conjugated anti-rabbit and anti-mouse secondary antibodies (1 : 5000 dilution). Proteins were visualized using ECL chemiluminescence agents and developed by autoluminography.

### 2.4. Statistical Analysis

All results are expressed as the means ± SD and analyzed by one-way analysis of variance (ANOVA) (SPSS version 17.0 software; SPSS Inc., Chicago, IL, USA). Quantitative data were tested for homogeneity of variance. If the variances were homogeneous, analysis of variance (ANOVA) followed by the post hoc least significant difference (LSD) test was used for multiple comparisons. If the variances were not homogeneous, a one-way ANOVA was performed followed by the Dunnett's T3 post hoc test for multiple comparisons. A *P* value of *<*0.05 was considered significant.

## 3. Results

### 3.1. SAA Attenuates Oxidative Stress in 5/6Nx Rats

**Figure 1:**
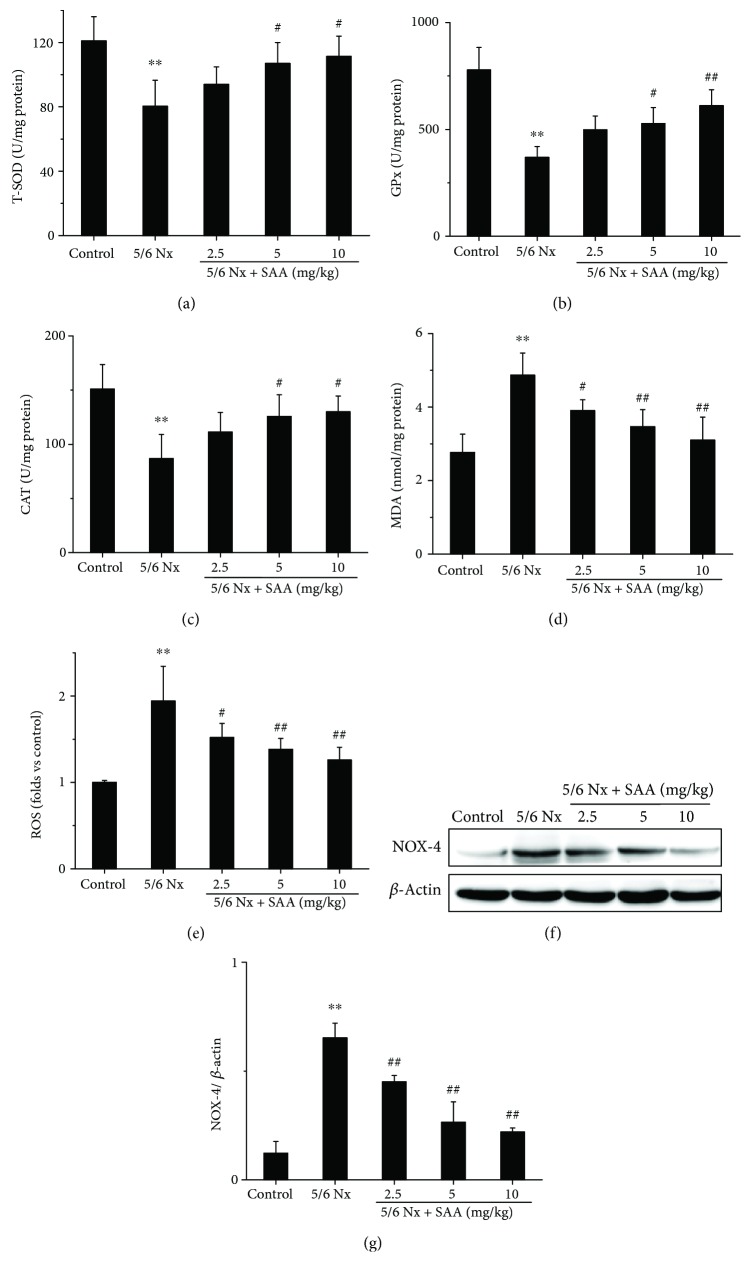
The effects of SAA on the activities of T-SOD, GPx, and CAT and the levels of MDA, ROS, and NOX-4 in 5/6Nx rats. The levels of (a) T-SOD activity, (b) GPx activity, (c) CAT activity, and (d) MDA were measured using commercial kits; (e) the level of ROS was determined by 2′,7′-dichlorofluorescein diacetate (DCFH-DA); (f) the expression of NOX-4 protein was analyzed by Western blot; (g) NOX-4 protein expression relative to protein *β*-actin. T-SOD represents total superoxide dismutase; GPx, CAT, MDA, ROS, SAA, and 5/6Nx stand for glutathione peroxidase, catalase, malondialdehyde, reactive oxygen species, salvianolic acid A, and 5/6 nephrectomized, respectively. Data are expressed as the means ± SD. ^∗∗^*P* < 0.01 vs the control group; ^#^*P* < 0.05 and ^##^*P* < 0.01 vs the 5/6Nx group.

The 5/6Nx rat model was successfully established in our previous study [33], in which all rats were subjected to 5/6 nephrectomy. To investigate the effects of SAA on oxidative stress in 5/6Nx rats, the levels of renal T-SOD, GPx, CAT activity, MDA, and ROS were tested. The results show that the levels of MDA and ROS were significantly increased in 5/6Nx rats compared to the control group, while the activities of T-SOD, GPx, and CAT were all decreased, which suggests that oxidative stress was grievous in this model (Figure 1). Administration of SAA significantly increased the activities of T-SOD, GPx, and CAT compared to the untreated 5/6Nx group and lowered the levels of MDA and ROS in a dose-dependent manner (Figure 1) suggesting that SAA plays a key role in this model.

In addition, the renal level of NOX-4, which is a major source of ROS formation in renal pathogenic conditions, was also analyzed [35]. The NOX-4 protein level was significantly increased in 5/6Nx rats compared to the control group rats. Treatment with SAA resulted in significantly lower protein expression of NOX-4 compared to the untreated 5/6Nx group in a dose-dependent manner (Figures 1(f) and 1(g)).

### 3.2. SAA Activates the Akt/GSK-3*β*/Nrf2 Signaling Pathway in 5/6Nx Rats

**Figure 2:**
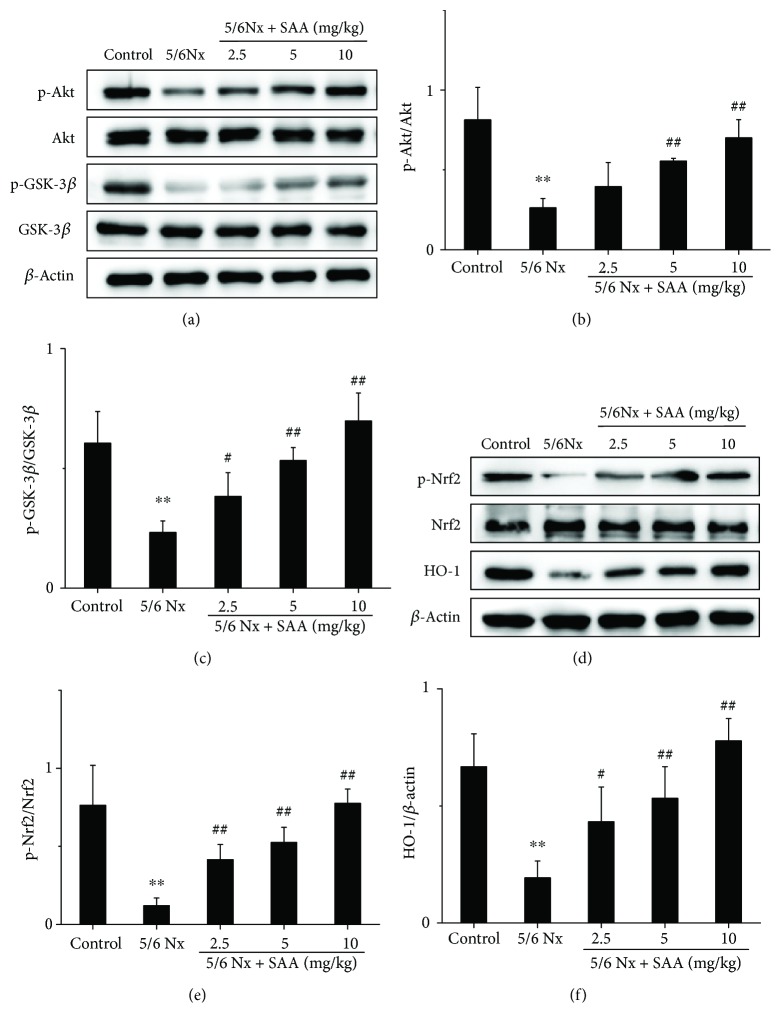
The effects of SAA on the Akt/GSK-3*β*/Nrf2 signaling pathway in 5/6Nx rats. (a) The protein expression levels of p-Akt, Akt, p-GSK-3*β*, and GSK-3*β* were analyzed by Western blot; (b) p-Akt protein expression relative to Akt protein levels; (c) p-GSK-3*β* protein expression relative to GSK-3*β* protein levels; (d) the protein expression levels of p-Nrf2, Nrf2, and HO-1 were analyzed by Western blot; (e) p-Nrf2 protein expression relative to Nrf2 protein levels; (f) HO-1 protein expression relative to *β*-actin protein expression. Data are expressed as the mean ± SD. ^∗∗^*P* < 0.01 vs the control group; ^#^*P* < 0.05 and ^##^*P* < 0.01 vs the 5/6Nx group.

Some studies suggested that the Akt/GSK-3*β*/Nrf2 signaling pathway is closely associated with kidney oxidative stress [28, 36]. Thus, major proteins of the Akt/GSK-3*β*/Nrf2 signaling pathway were measured during CKD. The protein levels of p-Akt, p-GSK-3*β*, p-Nrf2, and HO-1 in 5/6Nx rats were significantly decreased compared to the control rats, which suggests that the Akt/GSK-3*β*/Nrf2 signaling pathway was inhibited during renal oxidative stress. Treatment with SAA significantly increased the protein expression of p-Akt, p-GSK-3*β*, p-Nrf2, and HO-1 compared to the 5/6Nx group in a dose-dependent manner, which suggests that SAA enhances the activation of the Akt/GSK-3*β*/Nrf2 signaling pathway (Figure 2).

### 3.3. SAA Improves Cell Viability and Lowers Oxidative Stress in H_2_O_2_-Induced HK-2 Cells

**Figure 3:**
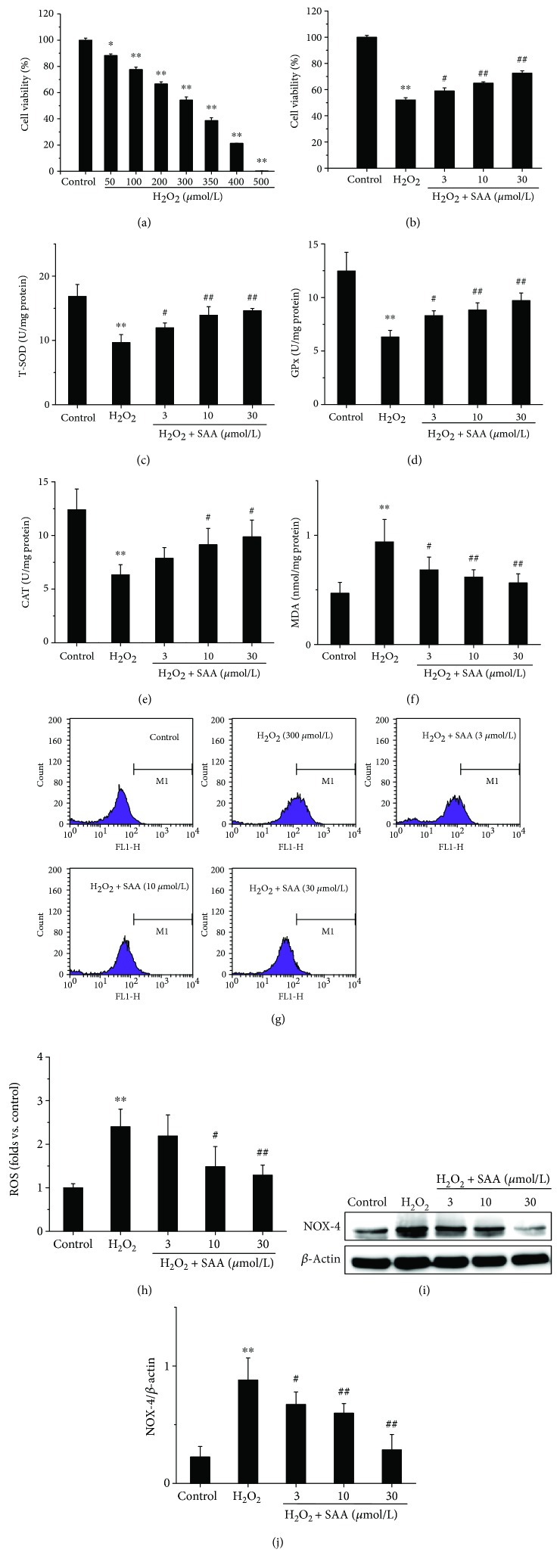
The effects of SAA on HK-2 cell viability and the levels of T-SOD, GPx, CAT activity, MDA, ROS, and NOX-4 in H_2_O_2_-induced HK-2 cells. Cells were pretreated with or without various concentrations of SAA for 2 h and then coincubated with or without H_2_O_2_ (300 *μ*mol/L) for the indicated time. (a) Effects of various concentrations of H_2_O_2_ on HK-2 cell viability and (b) effects of SAA on 300 *μ*mol/L H_2_O_2_-induced HK-2 cell viability were assessed by MTT assay. The levels of (c) T-SOD activity, (d) GPx activity, (e) CAT activity, and (f) MDA were measured using commercial kits; (g, h) the levels of ROS were determined by 2′,7′-dichlorofluorescein diacetate (DCFH-DA); (i) the expression of NOX-4 protein was analyzed by Western blot; (j) NOX-4 protein expression relative to protein *β*-actin expression. Data are expressed as the means ± SD (*n* = 3). ^∗^*P* < 0.05 and ^∗∗^*P* < 0.01 vs the control group; ^#^*P* < 0.05 and ^##^*P* < 0.01 vs the H_2_O_2_ group.

To further confirm the effects of SAA, we observed *in vivo* and we subsequently determined the effects of SAA in an *in vitro* model of kidney oxidative stress (H_2_O_2_-induced HK-2 cells). The results demonstrated that H_2_O_2_ inhibited the viability of HK-2 cells in a concentration-dependent manner and induced approximately 50% of cell viability loss at the concentration of 300 *μ*mol/L. Thereby, we selected a concentration of 300 *μ*mol/L H_2_O_2_ for our *in vitro* model. Although H_2_O_2_ had a cytotoxic effect on cells, treatment with SAA significantly enhanced the cell viability in a dose-dependent manner in H_2_O_2_-induced HK-2 cells (Figures 3(a) and 3(b)).

The levels of T-SOD, GPx, CAT activity, MDA, and ROS were also examined in H_2_O_2_-induced HK-2 cells. The activities of T-SOD, GPx, and CAT were all significantly decreased in H_2_O_2_-induced HK-2 cells compared to H_2_O_2_-untreated control cells, while the levels of MDA and ROS were increased, which indicates that the model of oxidative stress is functional *in vitro*. Administration of SAA significantly increased the activities of T-SOD, GPx, and CAT and attenuated the levels of MDA and ROS in H_2_O_2_-induced HK-2 cells in a concentration-dependent manner (Figures 3(c)–3(h)).

Furthermore, the expression of NOX-4 was also detected in H_2_O_2_-induced HK-2 cells. The NOX-4 protein level was significantly increased in H_2_O_2_-induced HK-2 cells compared to the control group. Treatment with SAA significantly lowered the level of NOX-4 protein in HK-2 cells stimulated with H_2_O_2_ in a concentration-dependent manner (Figures 3(i) and 3(j)).

### 3.4. SAA Enhances the Activation of the Akt/GSK-3*β*/Nrf2 Signaling Pathway in H_2_O_2_-Induced HK-2 Cells

**Figure 4:**
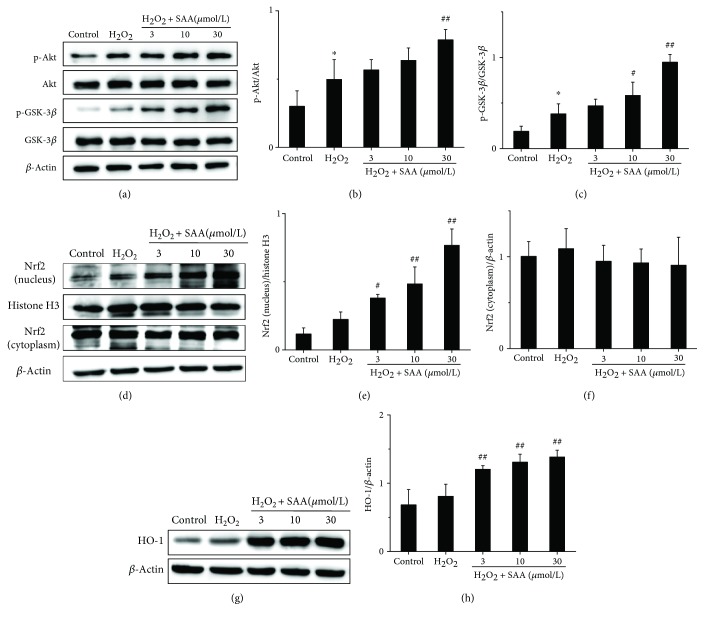
The effects of SAA on the Akt/GSK-3*β*/Nrf2 signaling pathway in H_2_O_2_-induced HK-2 cells. Cells were pretreated with or without various concentrations of SAA for 2 h and then coincubated with or without H_2_O_2_ (300 *μ*mol/L) for the indicated time. (a) The protein expression levels of p-Akt, Akt, p-GSK-3*β*, and GSK-3*β* were analyzed by Western blot; (b) p-Akt protein expression relative to Akt protein levels; (c) p-GSK-3*β* protein expression relative to GSK-3*β* protein levels; (d) the protein expression levels of nuclear Nrf2 and cytosolic Nrf2 were analyzed by Western blot; (e) nuclear Nrf2 protein expression relative to histone H3 protein expression; (f) cytosolic Nrf2 protein expression relative to *β*-actin protein expression; (g) the protein expression levels of HO-1 were analyzed by Western blot; (h) HO-1 protein expression relative to *β*-actin protein expression. Data are expressed as the means ± SD (*n* = 3). ^∗^*P* < 0.05 vs the control group; ^#^*P* < 0.05 and ^##^*P* < 0.01 vs the H_2_O_2_ group.

Major proteins of the Akt/GSK-3*β*/Nrf2 signaling pathway were also detected in H_2_O_2_-induced HK-2 cells. Stimulation with H_2_O_2_ upregulated the protein levels of p-Akt, p-GSK-3*β*, intranuclear Nrf2, and HO-1 compared to H_2_O_2_-untreated control cells (Figure 4), which may be relevant to the cell-adaptive response. However, the level of cytoplasmic Nrf2 remained relatively unaffected (Figures 4(d) and 4(f)). Treatment with SAA significantly increased the expression of p-Akt, p-GSK-3*β*, intranuclear Nrf2, and HO-1 proteins compared to HK-2 cells stimulated by H_2_O_2_ to varying degrees depending on the concentration of SAA. This suggests that SAA enhances the activation of the Akt/GSK-3*β*/Nrf2 signaling pathway (Figure 4). All of the *in vitro* results are consistent with the results obtained from the *in vivo* model using 5/6Nx rats.

### 3.5. SAA Partly Enhances the Activation of the Nrf2 Signaling Pathway through Inhibiting the NF-*κ*B Signaling Pathway in LPS-Induced HK-2 Cells

**Figure 5:**
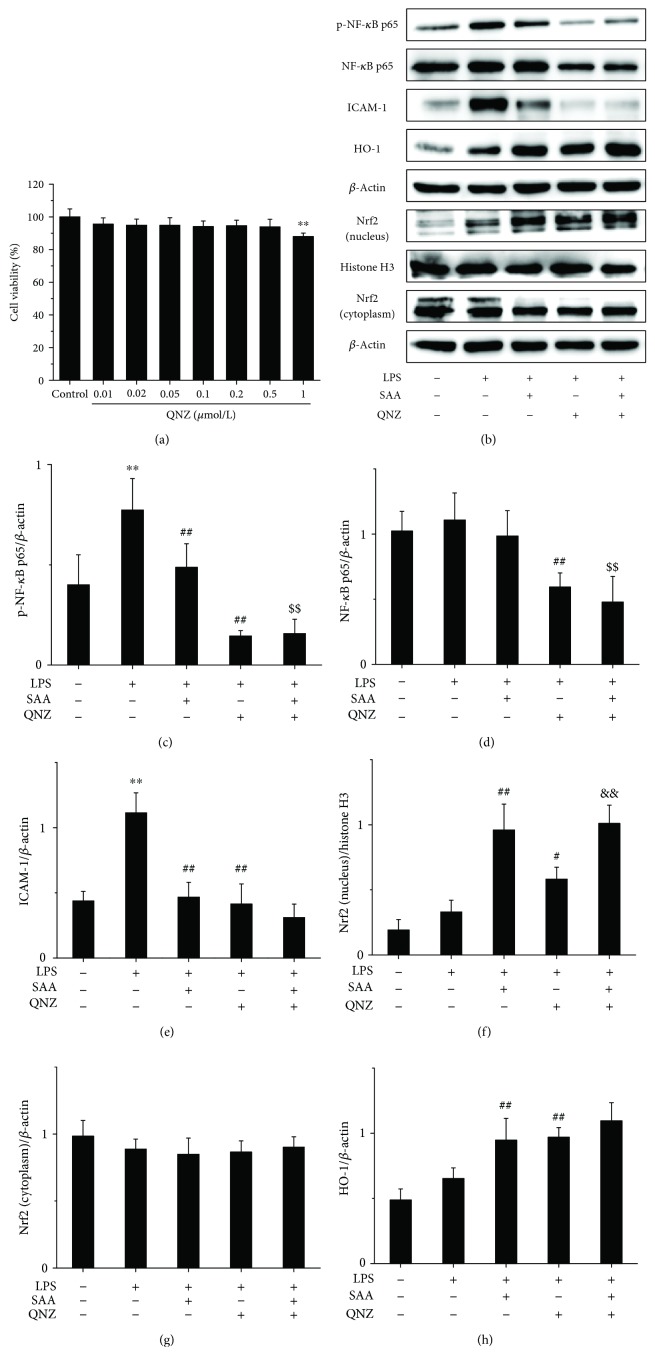
The effects of QNZ on HK-2 cell viability and SAA on the NF-*κ*B and Nrf2 signaling pathways in LPS-induced HK-2 cells. Cells were treated with or without various concentrations of QNZ for 24 h, and cell viability was assessed by MTT assay. Cells were pretreated with or without 0.5 *μ*mol/L QNZ for 10 h and then cotreated with or without 30 *μ*mol/L SAA for 2 h and lastly stimulated with or without LPS for 1.5 h. (a) Effects of QNZ on HK-2 cell viability; (b) the protein expression levels of p-NF-*κ*B p65, NF-*κ*B p65, ICAM-1, HO-1, Nrf2 (nucleus), and Nrf2 (cytoplasm) proteins were analyzed by Western blot; (c) p-NF-*κ*B p65 protein expression relative to *β*-actin protein expression; (d) NF-*κ*B p65 protein expression relative to *β*-actin protein expression; (e) ICAM-1 protein expression relative to *β*-actin protein expression; (f) Nrf2 (nucleus) protein expression relative to histone H3 protein expression; (g) Nrf2 (cytoplasm) protein expression relative to *β*-actin protein expression; (h) HO-1 protein expression relative to *β*-actin protein expression. Data are expressed as the means ± SD (*n* = 3). ^∗∗^*P* < 0.01 vs the control group; ^#^*P* < 0.05 and ^##^*P* < 0.01 vs the LPS group; ^$$^*P* < 0.01 vs the SAA group; ^&&^*P* < 0.01 vs the QNZ group.

To further study the relationship between the NF-*κ*B and Nrf2 signaling pathways, QNZ and ML385 were used *in vitro*. The results revealed that QNZ did not inhibit the viability of HK-2 cells up to a concentration of 0.5 *μ*mol/L and was cytotoxic to HK-2 cells at a concentration of 1 *μ*mol/L (Figure 5(a)). Thus, a concentration of 0.5 *μ*mol/L QNZ was selected for our *in vitro* model. Stimulation with LPS significantly increased the protein levels of p-NF-*κ*B p65 and ICAM-1 (Figures 5(b), 5(c) and 5(e)), which suggests that the NF-*κ*B signaling pathway was activated. Both SAA and QNZ significantly lowered the expression of p-NF-*κ*B p65 and ICAM-1 proteins (Figures 5(b), 5(c) and 5(e)), which suggests that SAA and QNZ inhibit the activation of the NF-*κ*B signaling pathway. Administration of QNZ alone also significantly increased the expression of intranuclear Nrf2 and HO-1 proteins compared to HK-2 cells stimulated with LPS (Figures 5(b), 5(f) and 5(h)), which indicates that NF-*κ*B inhibits the Nrf2 signaling pathway. Treatment with SAA significantly increased the expression of intranuclear Nrf2 and HO-1 proteins compared to HK-2 cells stimulated by LPS, which is enhanced by QNZ to some extent (Figures 5(b), 5(f) and 5(h)). Thus, SAA activates the Nrf2 signaling pathway indirectly through inhibiting the NF-*κ*B signaling pathway. Compared to the administration of QNZ, SAA increased the expression of intranuclear Nrf2 and HO-1 proteins in HK-2 cells stimulated by LPS (Figures 5(b), 5(f) and 5(h)), which suggests that SAA activates the Nrf2 signaling pathway directly. Thus, SAA does activate not only directly but also indirectly the Nrf2 signaling pathway based on the inhibition of NF-*κ*B.

### 3.6. SAA Partly Inhibits the Activation of the NF-*κ*B Signaling Pathway through Activating the Nrf2 Signaling Pathway in H_2_O_2_-Induced HK-2 Cells

**Figure 6:**
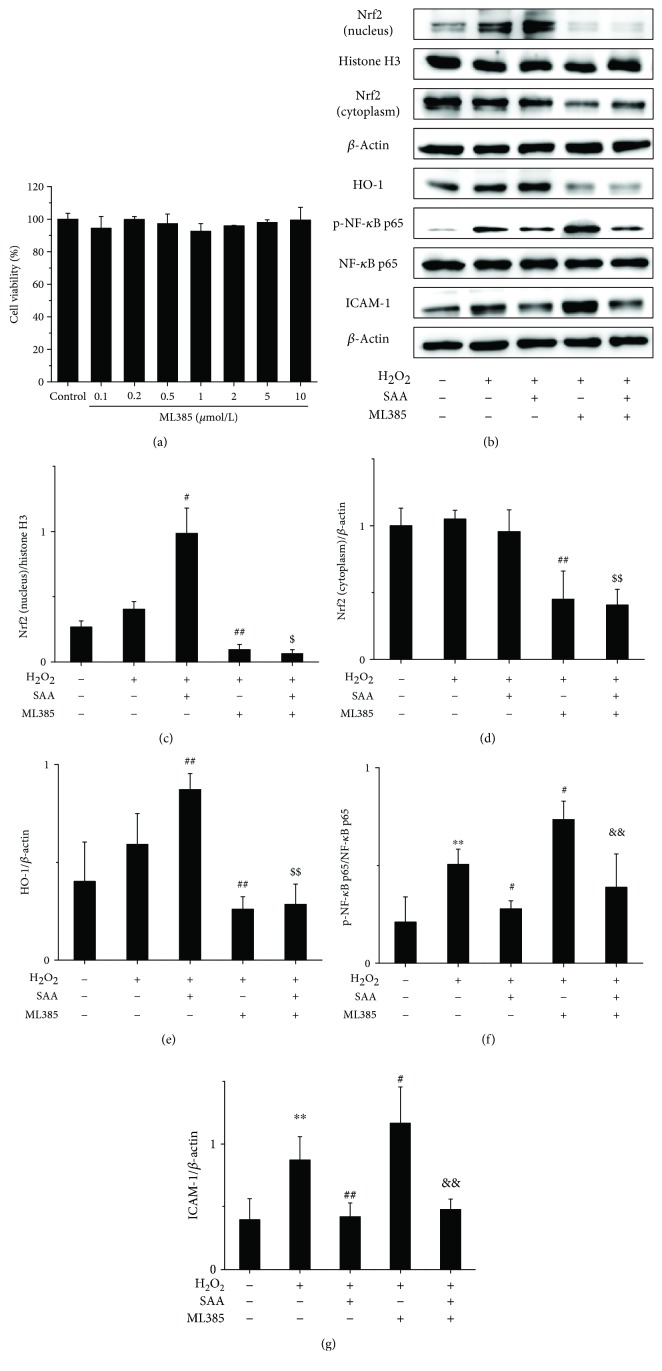
The effects of ML385 on HK-2 cell viability and SAA on the Nrf2 and NF-*κ*B signaling pathways in H_2_O_2_-induced HK-2 cells. Cells were treated with or without various concentrations of ML385 for 24 h, and cell viability was assessed by MTT assay. Cells were pretreated with or without 10 *μ*mol/L ML385 for 10 h and then cotreated with or without 30 *μ*mol/L SAA for 2 h and lastly stimulated with or without H_2_O_2_ for 4 h. (a) Effects of ML385 on HK-2 cell viability; (b) the protein expression levels of Nrf2 (nucleus), Nrf2 (cytoplasm), HO-1, p-NF-*κ*B p65, NF-*κ*B p65, and ICAM-1 proteins were analyzed by Western blot; (c) Nrf2 (nucleus) protein expression relative to histone H3 protein expression; (d) Nrf2 (cytoplasm) protein expression relative to *β*-actin protein expression; (e) HO-1 protein expression relative to *β*-actin protein expression; (f) p-NF-*κ*B p65 protein expression relative to NF-*κ*B p65 protein expression; (g) ICAM-1 protein expression relative to *β*-actin protein expression. Data are expressed as the means ± SD (*n* = 3). ^∗∗^*P* < 0.01 vs the control group; ^#^*P* < 0.05 and ^##^*P* < 0.01 vs the H_2_O_2_ group; ^$^*P* < 0.05 and ^$$^*P* < 0.01 vs the SAA group; ^&&^*P* < 0.01 vs the ML385 group.

The results revealed that ML385 did not inhibit the viability of HK-2 cells up to a concentration of 10 *μ*mol/L (Figure 6(a)). Therefore, 10 *μ*mol/L of ML385 was selected for our *in vitro* model. SAA significantly increased the expression of intranuclear Nrf2 and HO-1 proteins in H_2_O_2_-induced HK-2 cells (Figures 6(b), 6(c) and 6(e)), which suggests that SAA enhances the activation of the Nrf2 signaling pathway. However, ML385 decreased the protein levels of Nrf2 and HO-1 (Figures 6(b)–6(e)), suggesting that ML385 significantly inhibits the Nrf2 signaling pathway. Administration with ML385 alone significantly increased the expression of p-NF-*κ*B p65 and ICAM-1 proteins compared to HK-2 stimulated by H_2_O_2_ (Figures 6(b), 6(f) and 6(g)), which indicates that Nrf2 inhibits the NF-*κ*B signaling pathway. SAA significantly lowered the expression of p-NF-*κ*B p65 and ICAM-1 proteins compared to HK-2 cells stimulated by H_2_O_2_, which can be abrogated by ML385 to some degree (Figures 6(b), 6(f) and 6(g)). Thus, SAA inhibits the NF-*κ*B signaling pathway indirectly through activating the Nrf2 signaling pathway. Compared to the administration with ML385, SAA significantly lowered the expression of p-NF-*κ*B p65 and ICAM-1 proteins in H_2_O_2_-treated HK-2 cells (Figures 6(b), 6(f) and 6(g)), which shows that SAA inhibits the NF-*κ*B signaling pathway directly. Thus, SAA does inhibit not only directly but also indirectly the NF-*κ*B signaling pathway based on the activation of Nrf2.

## 4. Discussion

One of the hallmarks of CKD is the presence of oxidative stress characterized by a combination of increased ROS and impaired antioxidant capacity [5]. Some studies have shown that an increase of ROS production and an impaired activity of antioxidant enzymes occur using animal models of CKD [11, 15, 37, 38]. In this study, we adopted the 5/6 nephrectomy method to set up an experimental model of CKD, which shows pathological changes similar to those observed in CKD patients [39]. Therefore, this model is frequently used in the field to investigate the effects of pharmacological and other factors on functional and morphological changes in the kidney [40]. In our previous study, we have demonstrated that detrimental renal dysfunction and pathological lesions improved after SAA treatment in 5/6Nx rats [33], which is related to oxidative stress and inflammation. Liu et al. have reported that Shen-Kang protects 5/6 nephrectomized rats against renal injury by reducing oxidative stress [11], and another study reported that inhibiting oxidative stress would attenuate renal dysfunction [16]. As a water-soluble minor phenolic acid, SAA exhibited antioxidant activities in various animal disease models including kidney and liver injury and myocardial infarction [34, 41, 42]. In our current study, the results show that SAA decreased the levels of MDA, ROS, and NOX-4 and improved the activities of T-SOD, GPx, and CAT in 5/6Nx rats, suggesting that SAA might have a beneficial effect on kidney injury through its antioxidant activity (Figure 1).

**Figure 7:**
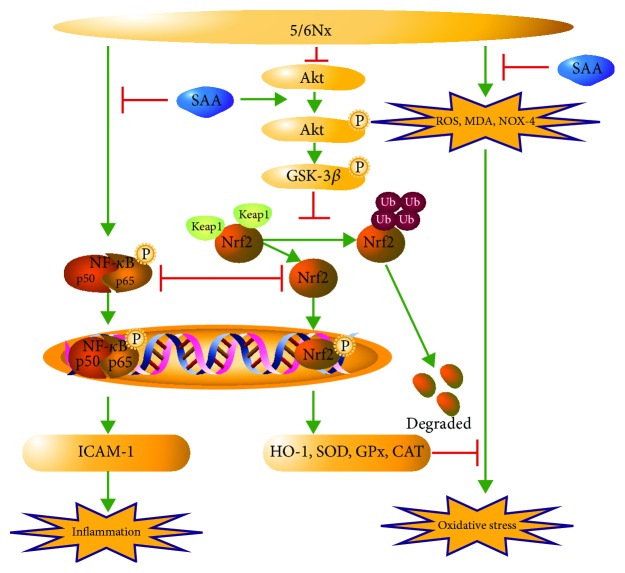
A schematic illustration of SAA attenuating kidney oxidative stress in 5/6Nx rats. SAA protects the kidney against oxidative stress through activating the Akt/GSK-3*β*/Nrf2 signaling pathway and inhibiting the NF-*κ*B signaling pathway in 5/6Nx rats.

It has been demonstrated that the impaired Nrf2 activity accelerated oxidative stress and inflammation in CKD rats and that treatment with an Nrf2 activator ameliorated kidney disease in experimental animal models [43]. Yoh et al. reported that ablation of the Nrf2 gene accelerates oxidative and nitrosative stress, inflammation, and renal injury in diabetic mice [44]. Given the importance of Nrf2 in CKD, we determined major proteins of the Nrf2/HO-1 pathway. In our present study, we investigated them in more detail and demonstrated that SAA inhibits oxidative stress by activating the Nrf2 signaling pathway and subsequently affects the expression of a series of downstream proteins (Figure 7). Our results show that SAA significantly increases the expression of p-Nrf2 and HO-1 proteins in 5/6Nx rats (Figures 2(d)–2(f)). Similar results were shown for HK-2 cells stimulated with H_2_O_2_ (Figure 4). It has been reported that Nrf2 activators, such as phenethyl isothiocyanate (PEITC), sulforaphane (SFN), and curcumin (CUR), attenuate the activation of the NF-*κ*B signaling pathway induced by lipopolysaccharide (LPS) [45]. This is consistent with our study which showed that SAA partly inhibited the activation of the NF-*κ*B signaling pathway through activation of the Nrf2 signaling pathway in HK-2 cells (Figure 6). Furthermore, SAA moderately enhanced the activation of the Nrf2 signaling pathway through inhibition of the NF-*κ*B signaling pathway in HK-2 cells (Figure 5). It was previously shown that the basic chemical structure of salvianolic acids is Danshensu, which exhibits wide cardiovascular benefits by activating the Nrf2 signaling pathway [46]. SAA consists of one molecule of Danshensu and two molecules of caffeic acid, which may explain the reason why SAA activates the Nrf2 signaling pathway.

In eukaryotes, the PI3K/Akt signaling pathway is widely expressed, which plays an essential role in proliferation, differentiation, survival, and apoptosis [47, 48]. Furthermore, this pathway is involved in the balance between the oxidative and antioxidative systems. It has been reported that the activation of PI3K/Akt decreases oxidative stress both *in vivo* and *in vitro* [27, 28, 49, 50]. There is one serine/threonine protein kinase downstream of Akt, GSK-3*β*, which is known to play important roles in many disorders such as oxidative stress, cancer, diabetes, psychiatric disorders, and Alzheimer's disease [51]. The phosphorylation of GSK-3*β* at Ser9 results in inactivation of GSK-3*β* by several kinases like Akt, protein kinase A (PKA), and PKC [51]. Studies have shown that the inhibition of GSK-3*β* lowers the level of nuclear Nrf2 through nuclear export and degradation of Nrf2 in liver cancer cells and improves cell survival during the later phases of oxidative stress [52, 53]. In our current study, we demonstrated that SAA increases the expression of p-Akt, p-GSK-3*β*, and p-Nrf2 in 5/6Nx rats (Figure 2). Similar results have been confirmed in H_2_O_2_-induced HK-2 cells *in vitro* (Figure 4). Our results indicate that SAA reduces oxidative stress through enhancing the activation of the Akt/GSK-3*β*/Nrf2 signaling pathway and increased the expression of its downstream proteins.

## 5. Conclusions

In conclusion, our results have demonstrated that SAA can effectively protect kidneys against oxidative stress in a 5/6Nx rat model. As shown in Figure 7, one of the pivotal mechanisms for the therapeutic effects of SAA on kidney injury was mainly related with its antioxidative roles by activating the Akt/GSK-3*β*/Nrf2 signaling pathway and inhibiting the NF-*κ*B signaling pathway, in which there was reciprocal repression between these two signaling pathways. In combination with our previous study [33], we can draw the conclusion that SAA effectively attenuates kidney injury of CKD, which can be attributed to its anti-inflammatory and antioxidative activities through inhibition of the NF-*κ*B and p38 MAPK signaling pathways and activation of the Akt/GSK-3*β*/Nrf2 signaling pathway. As a multifunctional agent, additional effects of SAA on CKD will be studied in more detail in the future. In summary, our novel findings in this study offer the pharmacological basis for exciting future studies of SAA for the treatment of CKD.

## Data Availability

The data used to support the findings of this study are available from the corresponding author upon request.
